# The Swedish Version of the Electronic Health Literacy Scale: Prospective Psychometric Evaluation Study Including Thresholds Levels

**DOI:** 10.2196/16316

**Published:** 2020-02-24

**Authors:** Josefin Wångdahl, Maria Jaensson, Karuna Dahlberg, Ulrica Nilsson

**Affiliations:** 1 Department of Public Health and Caring Sciences Uppsala University Uppsala Sweden; 2 Department of Neurobiology, Care Sciences and Society Karolinska Institute Huddinge Sweden; 3 School of Health Sciences Faculty of Medicine and Health Örebro University Örebro Sweden; 4 Department of Perioperative Medicine and Intensive Care Karolinska University Hospital Stockholm Sweden

**Keywords:** eHealth, literacy, internet, psychometrics

## Abstract

**Background:**

To enhance the efficacy of information and communication, health care has increasingly turned to digitalization. Electronic health (eHealth) is an important factor that influences the use and receipt of benefits from Web-based health resources. Consequently, the concept of eHealth literacy has emerged, and in 2006 Norman and Skinner developed an 8-item self-report instrument to measure these skills: the eHealth Literacy Scale (eHEALS). However, the eHEALS has not been tested for reliability and validity in the general Swedish population and no threshold values have been established.

**Objective:**

The aim of this study was to translate and adapt eHEALS into a Swedish version; evaluate convergent validity and psychometric properties; and determine threshold levels for inadequate, problematic, and sufficient eHealth literacy.

**Methods:**

Prospective psychometric evaluation study included 323 participants equally distributed between sexes with a mean age of 49 years recruited from 12 different arenas.

**Results:**

There were some difficulties translating the English concept *health resources*. This resulted in this concept being translated as *health information* (ie, *Hälsoinformation* in Swedish). The eHEALS total score was 29.3 (SD 6.2), Cronbach alpha .94, Spearman-Brown coefficient .96, and response rate 94.6%. All a priori hypotheses were confirmed, supporting convergent validity. The test-retest reliability indicated an almost perfect agreement, .86 (*P*<.001). An exploratory factor analysis found one component explaining 64% of the total variance. No floor or ceiling effect was noted. Thresholds levels were set at 8 to 20 = inadequate, 21 to 26 = problematic, and 27 to 40 = sufficient, and there were no significant differences in distribution of the three levels between the Swedish version of eHEALS and the HLS-EU-Q16.

**Conclusions:**

The Swedish version of eHEALS was assessed as being unidimensional with high internal consistency of the instrument, making the reliability adequate. Adapted threshold levels for inadequate, problematic, and sufficient levels of eHealth literacy seem to be relevant. However, there are some linguistic issues relating to the concept of *health resources*.

## Introduction

Globally, the internet is an important resource for health-related information and health services, which requires a range of digital skills among users and also new ways of describing and evaluating users’ digital capabilities and experience in this rapidly changing health context [1]. The use of the internet in Sweden has steadily increased in recent years, and currently about 95% of all households have internet access. Internet access has increased the most in the elderly population, and in people aged 76 years and older, 87% have internet access at home. Of those, 49% use the internet to seek health-related information, compared with 96% of persons aged 26 to 45 years, 90% to 95% of persons aged 46 to 65 years, and 76% of persons aged 66 to 75 years [2]. In the present health care system, people are expected to participate and be engaged in their own care; they must be able to understand health instructions regarding how to manage their care (ie, health literacy) [3-5]. Traditional health literacy refers to an individual’s ability to use printed information. However, with the increasing digitalization of information and services, the modern health care system must be aware of the health literacy levels of its patients in cyber space in order to maximize the benefits of electronic health (eHealth) technologies, challenging for both patients and health care staff [6].

The internet is significantly impacting health and health care, and it has the potential to advance health care delivery and support decision-making [1,7]. Thus, internet-enabled health care is a strategic priority globally. EHealth is an important factor that influences the use and receipt of benefits from Web-based health resources [1,8]. Consequently, the concept of eHealth literacy has emerged [1,9,10] and has been described as “the ability to seek, find, understand, and appraise health information from electronic sources and apply the knowledge gained to addressing or solving a health problem” [10].

Because eHealth literacy is a more recent construct than health literacy, it is hard to find studies that have been published regarding its association with health outcomes [11]. However, limited health literacy has been found to affect a person’s quality of care, resulting in lower satisfaction with care and a lower understanding of their medical situation [12,13]. This increases, for example, the probability of an adverse medication reaction because of misunderstanding the instructions [12,14]. Health literacy is also associated with the extent to which people benefit from health examinations [15], the quality of their postoperative recovery [16], and even mortality [11].

In a systematic review of questionnaires measuring eHealth literacy, 8 questionnaires were identified. It is noteworthy that the eHealth Literacy Scale (eHEALS) questionnaire was used in 45 of the 53 included articles [17]. eHEALS was developed in 2006 by Norman and Skinner [10] and aims to measure a broad range of literacy skills, which could make it useful in assessing the effects of strategies for delivering online information and applications. eHEALS is an 8-item instrument with each item scored on a 5-point Likert scale with response options ranging from strongly agree to strongly disagree. Total scores on the eHEALS range from 8 to 40, with higher scores representing higher self-perceived eHealth literacy [10].

The eHEALS is available in a range of languages [9,10,18-23], and the English version has been successfully administered via telephone [24]. Psychometric testing of eHEALS indicates that it is a reliable and valid instrument [10,23,25-27] but also that its validity requires further investigation [9]. However, there are no threshold levels for eHEALS, and eHEALS has not been tested for validity in the general Swedish population. Thus, the aim of our research was to translate and adapt the eHEALS into a Swedish version; evaluate convergent validity and psychometric properties; and determine threshold levels for inadequate, problematic, and sufficient eHealth literacy.

## Methods

### Study Design and Participants

This prospective psychometric evaluation study was conducted in three phases: translation, content validity testing, and psychometric evaluation. Data collection for phases 1 and 2 was completed from September 2018 to January 2019 and for phase 3 from February 2019 to May 2019 [28]. The project was approved by the Regional Ethical Review Board in Stockholm, Sweden, (no 2019/5:1) and follows the principles outlined in the 1964 Helsinki Declaration and its subsequent amendments. Participants received written and verbal information about the study, including its purpose and procedures, the voluntary nature of participation, and their option to withdraw at any time. By answering the questionnaire, participants consented to taking part in the study. Participants were also guaranteed confidentiality and secure data storage.

### Phase 1: Translation

Permission to translate and use the eHEALS [10] was obtained from the creator of the instrument, Cameron D Norman, PhD. After permission was granted, one professional translator with Swedish as a native language translated the original English version of eHEALS into Swedish (ie, the Swedish version of eHEALS [Sw-eHEALS]). The translator was instructed to use plain language and that the translation should be comprehensible to a 12-year-old child. This means that items should be short and simple and should not contain difficult words or jargon [29]. Two of the researchers (JW and UN) compared Sw-eHEALS with the original English version by examining how well it fit into the Swedish context and checking it for plain language. The researchers found that the Swedish version required some minor contextual changes and changes into simpler language in order to make it easier to understand the content. The translator stated that translating the English concept of *health resources* into Swedish was problematic because the Swedish concept of *Hälsoresurser* does not have the same meaning and the word is not commonly used in Swedish. The creator was contacted to discuss this, and he stated that there have been similar problems with the concept when translating it into other languages. Based on discussions with Dr Norman and also with four bilingual native English and Swedish speakers, it was decided to translate *health resources* as *Hälsoinformation* (ie, *health information*).

An expert panel including seven Swedish speakers was recruited to examine the quality of the translation [30]. The panel included two teachers of Swedish for people with a different mother tongue, two development managers with expertise in communication in health care, and three researchers in medicine, caring sciences, and health literacy. The experts were asked to comment on spelling, grammar, and whether they thought the translation had been written in plain language. After reviewing the experts’ feedback, the two researchers made some linguistic modifications and the Sw-eHEALS was then backtranslated by another native English-speaking translator who was blinded to the original eHEALS version. The backtranslated version and the original English eHEALS version were then compared by the translators and the two researchers. The two versions were found to match in terms of purpose and content.

### Phase 2: Face Validity

In order to evaluate the face validity [29,31] of the Sw-eHEALS, interviews were conducted with six participants recruited purposively and through snowball sampling [32] by two of the researchers (JW and UN) and one research assistant. A mix of ages, sexes, and educational levels was sought (see demographic characteristics of the participants in Table 1). Participants received verbal and written information about the face validity test and the main study and were instructed to think aloud during completion of the Sw-eHEAL and highlight any problematic points. They were also asked to reflect on why they selected specific responses.

Participants found the items easy to understand and answer and their verbal answers agreed with their marked answers in the Sw-eHEALS. There were no signs of misunderstandings. However, the concept of health information was interpreted slightly differently, even though the concept is broad. Some participants reported that the questions were quite similar and that they could be placed in a different order. They also reported that the Likert scale could include fewer or different alternatives. The face validity testing resulted in some minor changes in wording and confirmed the clarity and comprehensibility of Sw-eHEALS.

**Table 1:** Demographics of the content validity test group (n=6).

Variable		Value
**Gender**		
	Male	3
	Female	3
**Age in years**		
	Mean	50
	Range	28-78
**Educational level**		
	7-9 years	1
	10-12 years	2
	More than 12 years	3
Country of birth, Sweden		6

### Phase 3: Psychometric Evaluation

#### Participants and Settings

A study population comprising 300 participants was considered to be appropriate given that the general rule of thumb for factor analysis is 300 cases [33]. The inclusion criteria for participation was being an adult (aged 18 years and older), having Swedish as a native language, and being available on the day of the data collection. Participants were recruited from university courses, craft training, larger workplaces with academic and nonacademic staff, nongovernmental organizations serving elderly people, athletic clubs, and two choirs. A total of 12 arenas selected for diversity in age, sex, and level of education were visited by one of the researchers (JW).

#### Study Questionnaires and Additional Questions

The Sw-eHEALS, an additional questionniare, and general and demographics questions (age, biological sex, education level) were used. The HLS-EU-Q16 (Health Literacy Survey European Questionnaire, 16-item) aims to measure comprehensive health literacy (ie, perceived personal skills in finding, understanding, judging, and applying health information in order to maintain and improve health) [34]. The HLS-EU-Q16 was used to assess construct validity. HLS-EU-Q16 items were answered on a 4-point Likert scale ranging from very difficult to very easy. The total score of the index is summed to range from 0 to 16, with higher scores representing higher self-perceived comprehensive levels of health literacy. Score points between 0 to 8 represents inadequate health literacy, 9 to 12 score points represents problematic comprehensive health literacy, and 13 to 16 score points represents sufficient comprehensive health literacy [34,35].

One question was asked about general self-perceived health: “How do you assess your overall health status?” Response options were very poor, poor, fair, good, and very good [15,36,37].

Two questions were asked about interest in using the internet. “How useful is the internet in helping you make decisions about your health?” Response options to this *usability of the internet* question were not useful at all, not useful, unsure, useful, and very useful. “How important is it for you to be able to access health resources on the internet?” Response options to this *importance of the internet* question were not important at all, not important, unsure, important, and very important [10].

One question was asked about the frequency of internet use: “How often do you use the internet?” Response options were almost every day, several days a week, around one day a week, less than one day a week, and almost never [9].

#### Data Collection

On the day of the data collection, one of the researchers (JW) visited the arenas and informed participants verbally and in writing about the project and the meaning of informed consent. Those participants who agreed to participate answered the questionnaire directly. In one of the arenas, however, the organization manager distributed the written information and questionnaire instead of the researcher because it was difficult for all the staff to attend a meeting.

For analysis test-retest reliability, some of the participants were invited to answer the questionnaire twice within one week. A sample size in the retest of 25 participants was considered appropriate [38]. However, in order to include participants of different ages, sex, and education levels, 35 persons were asked to participate in the test-retest. In order to compare answers from the test and retest on an individual level and to ensure anonymity, participants marked their questionnaires with a code comprising the first three letters of their mother’s name and the year she was born.

#### Psychometric Testing

Psychometric testing was guided by the Consensus-Based Standards for the Selection of Health Measurement Instruments (COSMIN) [29,31,39].

##### Feasibility

Feasibility of the instrument was assessed by successful response rate and missing data from the questionnaires [39].

##### Construct Validity

Construct validity focuses on evaluating tests of the hypotheses and can be described as the degree to which scores of an instrument are consistent with a hypothesis [31]. Based on previous studies on health literacy showing positive associations between limited health literacy and high age [3,13,40,41], poor health [15,16,40,42,43], and low education level [41,44,45], hypotheses regarding correlations between Sw-eHEALS and age, level of education, and self-perceived general health were used. Hypotheses regarding positive correlations between Sw-eHEALS and interest in and level of internet use [9] were also used. Furthermore, positive correlations were seen between Sw-eHEALS and the HLS-EU-Q16 total score and the four HLS-EU-Q16 items measuring aspects of health literacy in relation to the internet [28].

#### Reliability

##### Internal Consistency

Internal consistency describes the degree of interrelatedness among items [31]:

- Exploratory factor analysis with principal axis factoring was used to identify the underlying relationships between the items in Sw-eHEALS [29].
- Cronbach alpha was calculated for the sum score and each item to assess the average correlation of items within each scale.
- Split-half reliability was used to measure the correlation between random split segments and determine how much error in a test score is due to poor test construction [46].

##### Test-Retest Reliability

Test-retest reliability can be described as the extent to which scores for the same participants are the same in measurements repeated over time [31].

##### Floor and Ceiling Effects

Floor and ceiling effects (ie, number of respondents who achieved the lowest or highest possible scores [29]) were examined. Floor or ceiling effects were considered a problem if more than 15% of a study population achieved the lowest or highest possible score [29].

##### Thresholds

The Sw-eHEALS scores were categorized according to the threshold values for health literacy assessed by the HLS-EU-Q16 [34,47]: inadequate = 0 to 8 (represents 50% of the sum score for HLS-EU-Q16), problematic = 9 to 12 (represents 25% of the sum score for HLS-EU-Q16), and sufficien*t* = 13 to 16 (represents 25% of the sum score for HLS-EU-Q16). Adapted to Sw-eHEALS scores, the thresholds for eHealth literacy are inadequate = 8 to 20 (represents 50% of the sum score for Sw-eHEALS), problematic = 21 to 26 (represents 25% of the sum score for Sw-eHEALS), and sufficien*t* = 27 to 40 (represents 25% of the sum score for Sw-eHEALS).

#### Statistical Analysis

Data are presented as mean, standard deviation, number, percentage, or range. Spearman rank was used to analyze the correlation between the total mean scores on Sw-eHEALS and HLS-EU-Q16. Self-perceived health, level of education, and age were also used. A coefficient magnitude of >.40 was considered evidence of construct validity (ie, moderate to strong correlations) [39]. Internal consistency was measured using a Spearman-Brown coefficient with values between .70 to .90 considered acceptable [48,49] and Cronbach alpha with a range of .70 to .95 considered acceptable [29,46]. Test-retest reliability was measured using the weighted kappa coefficient, with an accepted value of ≥.70 [29,50]. The Friedman test was used to analyze differences between Sw-eHEALS and HLS-EU-Q16 in terms of numbers of patients with inadequate, problematic, and sufficient health literacy. The chi-square test was used to analyze differences in sex, Student *t* test was used to analyze differences in age, and the Wilcoxon signed-rank test was used to analyze differences in age, educational levels, general self-perceived health, and Sw-eHEALS levels between participants with the same levels of health literacy on both the Sw-eHEALS and HLS-EU-Q16 compared with those with different levels. All data were analyzed using SPSS Statistics version 24.0 for Windows (IBM Corp). Two-tailed *P* values less than .05 were considered significant.

## Results

### Feasibility

**Figure 1:**
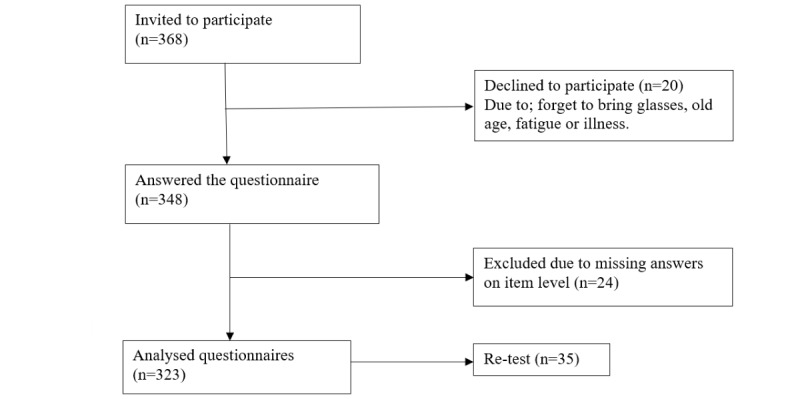
Flowchart of the data collection.

A total of 368 persons were invited to participate, and 348 answered the study questionnaires, giving a response rate of 94.6%; 24 questionnaires were incomplete and were excluded, resulting in a total of 323 valid questionnaires included in the analysis (Figure 1). There were no statistically significant differences regarding sex, age, or highest education level between the included participants versus those who declined to participate. Also, no pattern of structural problems in terms of difficulties in responding to certain items was found.

Sex was equally distributed, and the mean age was 49.2 (SD 21.5) years ranging from 19 to 94 years. Of the total, 90.4% (292/323) had at least 10 years’ education, and 85.8% (277/323) perceived their own general health as being good or very good. The majority (231/323, 71.5%) had sufficient comprehensive health literacy (HLS-EU-Q16), and the mean sum score of Sw-eHEALS was 29.3. Most participants reported that they used the internet almost every day (284/323, 87.9%), that they thought the internet was useful or very useful (243/323, 75.2%), and that the internet was important or very important (250/323, 77.4%; Table 2).

**Table 2:** Demographics of the respondents with a valid eHEALS sum score (n=323) and the test-retest group (n=35).

Characteristics			All		Test-retest group
**Biological sex, n (%)^a^**					
	Man	160 (50)		21 (60)	
	Woman	160 (50)		14 (40)	
**Age in years**					
	Mean (SD)	49.2 (21.5)		44.4 (12.2)	
	Range	19-94		26-89	
**Highest education level, n (%)**					
	1-6 years	4 (1)		1 (3)	
	7-9 years	24 (8)		2 (6)	
	10-12 years	149 (47)		4 (11)	
	Graduated from university	143 (45)		28 (80)	
**General self-perceived health, n (%)**					
	Very poor	0 (0)		0 (0)	
	Poor	8 (3)		1 (3)	
	Fair	38 (12)		1 (1)	
	Good	197 (61)		25 (71)	
	Very good	80 (25)		8 (23)	
**HLS-EU-Q16^b^, n (%)**					
	Inadequate	20 (6)		2 (6)	
	Problematic	72 (22)		4 (12)	
	Sufficient	231 (72)		27 (82)	
**Sw-eHEALS^c^**					
	Mean (SD)	29.3 (6.2)		31.2 (33.0)	
	Range	8-40		12-40	
**Frequency of internet use, n (%)**					
	Almost never	8 (3)		1 (3)	
	Less than 1 day a week	1 (0)		0 (0)	
	Around 1 day a week	8 (3)		1 (3)	
	Several days a week	22 (7)		2 (6)	
	Almost every day	284 (88)		31 (89)	
**Usability of the internet, n (%)**					
	Not useful at all	12 (4)		1 (3)	
	Not useful	12 (4)		2 (6)	
	Unsure	52 (16)		1 (3)	
	Useful	171 (54)		17 (49)	
	More useful	72 (23)		14 (40)	
**Importance of the internet, n (%)**					
	Not important at all	11 (3)		1 (3)	
	Not important	16 (5)		3 (9)	
	Unsure	43 (13)		2 (6)	
	Important	137 (43)		15 (43)	
	Very important	113 (35)		14 (40)	

**^a^**Missing n=3.

**^b^**HLS-EU-Q16: Health Literacy Survey European Questionnaire, 16-item.

**^c^**Sw-eHEALS: eHealth Literacy Scale.

### Construct Validity

The Sw-eHEALS sum score was weak and negatively correlated with age and weak and positively correlated with education level, self-perceived health, frequency of using the internet, and two items in the HLS-EU-Q16. Moderate positive correlations were found with perceptions of the internet as being useful and important, the HLS-EU-Q16 sum score, and two items on the HLS-EU-Q16 (Table 3).

**Table 3:** Spearman rho correlations between the Sw-eHEALS sum score and demographic characteristics, questions, and questionnaires.

Variable	Value	*P* value
Age	–0.30	<.01
Education level	0.23	<.05
Self-perceived health	0.19	<.01
Usability of the internet	0.57	<.05
Importance of the internet	0.47	<.05
Frequency of internet use	0.36	<.05
HLS-EU-Q16^a^ sum score	0.47	<.05
HLS-EU-Q16 item: Finding information about the treatment of illnesses that concern you	0.51	<.05
HLS-EU-Q16 item: Assessing whether informa­tion on health risks in the media is reliable	0.49	<.05
HLS-EU-Q16 item: Deciding on how you can protect yourself from illness based on information in the media	0.37	<.05
HLS-EU-Q16 item: Understanding informa­tion in the media about how to get healthier	0.38	<.05

^a^HLS-EU-Q16: Health Literacy Survey European Questionnaire, 16-item.

### Reliability

Factor analysis showed that the Kayser-Meyer-Olkin measure of sampling adequacy for the analysis was good (92, *P*<.001). The eigenvalue was 5.5 and explained 69% of the total variance, also reflected in the scree plot, which supported a unidimensional scale. All items loaded high ranging from .73 to .86. Cronbach alpha for the sum score of Sw-eHEALS was .94 and ranged from .92 to .93 for the individual items. The Spearman-Brown coefficient for the sum score of Sw-eHEALS was .96. Weighted Cohen kappa coefficient was acceptable for the sum score (.86, *P*<.001) and ranged from .64 to .79 (*P*<.001) for the individual items (Table 4).

**Table 4:** Reliability testing: exploratory factor analysis, Cronbach alpha, Spearman-Brown coefficient, and weighted quadratic Cohen kappa for the Swedish version of the eHealth Literacy Scale sum score or individual items.

Variable	Exploratory factor analysis	Cronbach alpha	Spearman-Brown coefficient	Weighted quadratic Cohen kappa
Sw-eHEALS^a^ total score	—	.94	.96	.86
Item 1: I know what health resources are available on the internet	.73	.93	—	.64
Item 2: I know where to find helpful health information on the internet	.83	.93	—	.71
Item 3: I know what health information is available on the internet	.86	.92	—	.70
Item 4: I know how to find helpful health information^b^ on the internet	.85	.92	—	.79
Item 5: I know how to use the health information^b^ I find on the internet to help me	.82	.93	—	.72
Item 6: I have the necessary skills to evaluate the health resources I find on the internet	.74	.93	—	.75
Item 7: I can distinguish between high- and low-quality health information on the internet	.78	.93	—	.68
Item 8: I feel confident in using information from the internet to make health decisions	.79	.93	—	.72

^a^Sw-eHEALS: Swedish version of the eHealth Literacy Scale.

^b^Health information=health resources in the original version by Norman and Skinner [10]

#### Test-Retest Reliability

A total of 35 participants were included in the test-retest. The mean age was 44 years with a range of 26 to 89 years, 60% (21/35) were male, 91% (32/35) had at least 10 years’ education and 94% (33/35) perceived their own general health as being good or very good. The majority (82%, 27/35) had sufficient comprehensive health literacy (HLS-EU-Q16), and the mean sum score of the Sw-eHEALS was 32.1. Most participants reported that they used the internet almost every day (89%, 31/35), that they thought the internet was useful or very useful (89%, 31/35), and that the internet was important or very important (83%, 29/35; Table 2). The weighted quadratic Cohen kappa for the Sw-eHEALS total score was .86 (*P*<.001) and ranged from .70 to .79 (*P*<.001) for 6 items and .64 to .68 (*P*<.001) for 2 items (Table 4).

#### Floor and Ceiling Effects

A total of 2% (7/323) of the participants had the lowest possible sum score and 4% (15/323) the highest possible sum score on the Sw-eHEALS.

#### Thresholds

The thresholds adapted for Sw-eHEALS resulted in 7.1% (23/323) of participants with inadequate, 18.8% (61/323) with problematic, and 74.0% (239/323) with sufficient eHealth literacy. When comparing numbers of participants with inadequate, problematic, and sufficient health literacy between Sw-eHEALS and HLS-EU-Q16, there were no statistical differences (*P*=.10), indicating that the thresholds determined for Sw-eHEALS seem to be relevant. Distribution between the three levels of eHealth literacy was similar for the HLS-EU-Q16, with 6.2% (20/323) of participants with inadequate, 22.3% (72/323) with problematic, and 71.5% (231/323) with sufficient eHealth literacy (Table 2). When dichotomized into insufficient (inadequate + problematic) and sufficient eHealth literacy, there was a significantly greater proportion of participants who scored the same levels of health literacy on both questionnaires (46+208=254/323, 78.6%) compared with participants who had different scores (42+27=69/323, 21.4%; *P*<.001; Table 5). There were no significant differences in age, sex, educational level, or general self-perceived health between these two groups.

**Table 5:** Distribution of participants scoring insufficient and sufficient health literacy and eHealth literacy.

	Insufficient health literacy, n (%)	Sufficient health literacy, n (%)
Insufficient electronic health literacy, n (%)	46 (14.3)	27 (8.3)
Sufficient electronic health literacy, n (%)	42 (13.1)	208 (64.3)

## Discussion

### Principal Findings

The results of this study support the intended use of Sw-eHEALS for measuring the self-reported eHealth literacy of Swedish persons. This paper shows that the process of translating an instrument from English into Swedish is not simple and quick. Capturing the culture and meaning of the words can be a challenge. Finding a suitable term for *health resources* in Swedish was problematic. Thus, several steps were taken that involved contacting the creator of the original instrument and also discussing the issue with experts and laymen. It is important that the content of the items remains the same as in the original. This is why we have described the translation process thoroughly. Although it is important that the content of the items remains the same as in the original version and reflects the true meaning of the construct, the wording or word order in the translated versions must be suitable for the target language and understandable by speakers with different levels of education and health literacy [30,51].

It has also been emphasized that translated items can assume different meanings and can affect the meanings perceived by the respondents. A lot of problems stem from the fact that the questions in the questionnaire or the wording of items in the instrument are culturally embedded. In other cases, structural differences mean that the exact equivalent objects or entities do not exist or that terms used to describe something in one country describe something else in another [52]. In this study, Sw-eHEALS was perceived as being easy to understand and answer, and no structural problems with specific items were found.

Our study found 1-factor structure (ie, unidimensionality) of the Sw-eHEALS, which is in line with previous studies, irrespective of using classical or modern test theory, as well as in different languages and populations [9,18,20,22,53-56]. The unidimensionality indicates that all the items measure a single underlying construct that is in line with what was originally proposed by the authors of the instrument [10]. However, a 2-factor structure has been reported [26,57,58], divided into the constructs of knowledge about resources and evaluation of resources. A 3-factor structure has also been reported including the construct: awareness, skills, and evaluation [8,59]. The 1-factor structure is important to Sw-eHEALS because this indicates it is appropriate to sum the item scores into a total score.

Internal consistency was assessed using Cronbach alpha and split-half reliability; both these coefficients were high, and the published recommendations for Cronbach alpha (ie, .70 to .95) were satisfied [29]. Other language versions of eHEALS have also reported high reliability with Cronbach alpha ≥.88 [18,20,22,53-55]. A high Cronbach alpha is usually found for questionnaires that contain a large number of items because Cronbach alpha is dependent on the number of items in a questionnaire [29]. The Sw-eHEALS includes 8 items.

The construct validity of the Sw-eHEALS was acceptable. Moderate positive correlations were found with the HLS-EU-Q16, which is in line with Neter et al [58]. Moderate positive correlations were also found with perceptions of the internet as being useful and important for finding information about the treatment of illnesses that cause concern and assessing whether information about health risks in the media is reliable. Previous studies support the relationship between eHealth literacy, use of the internet [9,18,20,53,60], mobile phone use [20], computer knowledge [53], and the amount of time spent online [20,54]. The findings have suggested that frequent internet users use the internet for health reasons and that this could result in a great level of self-reported eHealth literacy and that frequent internet users perceive that their ability to engage with and evaluate general internet resources is transferable to health-related content [8]. Furthermore, low eHealth literacy levels appear to be associated with poor skills using a personal computer, downloading files, and finding health information online and difficulties in receiving help from online sources [20].

The adapted threshold levels for inadequate, problematic, and sufficient levels of eHealth literacy were based on the levels for the HLS-EU-Q16 [35,47]. The threshold levels for Sw-eHEALS seem to be relevant, and it is important to establish these levels in order to identify those individuals and groups who suffer from inadequate and problematic eHealth literacy and are in need of support. To use a questionnaire without any thresholds or cutoff levels in research or in clinical practice is problematic because it is hard to evaluate what the values reflect (ie, insufficient or sufficient eHealth literacy). However, these suggested threshold levels for eHEALS have to be further evaluated in other populations and in other languages.

The test-retest reliability for the Sw-eHEALS sum score was .86, indicating an almost perfect agreement [50], to be compared with the creators of eHEALS, *r*=.68 [10], and the Persian version, *r*=.85 [55], analyzed using Pearson correlation. In our study, the time period between repeated measurements was one week, compared with the Persian version, which had a 2-week time period [55], and the original version, which had a 6-month follow-up [10]. If the time between the two tests is too long, respondents could have been exposed to things that changed their opinions, feelings, or attitudes about their behavior [51]. Terwee et al [29] believe that the time period between repeated administrations should be long enough to prevent recall but short enough to ensure that clinical changes have not occurred. Often, one or two weeks will be sufficient.

Floor and ceiling effects were acceptable; 2% of the participants scored the worst possible score (8), and 4% scored the best possible score (40). An acceptable floor and ceiling effect of eHEALS has been reported for the Italian version [18] and the Dutch version [9] in persons suffering from chronic disease [22] and in persons with moderate to high cardiovascular risk [26]. In an ideal situation, a questionnaire should be able to measure the entire spectrum of a phenomenon. If floor or ceiling effects are present, it is likely that extreme items will be missing at the lower or upper end of the scale. As a consequence, people with the lowest or highest possible score cannot be distinguished from each other, reducing reliability [29]. However, it has been reported that eHEALS does not seem to be able to detect small but clinically important changes in participants with mid to higher levels of eHealth literacy in a population suffering from moderate to high cardiovascular risk [26].

### Limitations

This study has a number of limitations. One limitation is that the sample included may not be representative of the majority of Swedish speakers. However, the included participants were recruited from different arenas, including groups of different ages, sex, and levels of education. Second, eHEALS measures self-reported eHealth literacy, which is not the same as measuring the person’s knowledge of eHealth. Self-reported eHealth literacy might be over- or underestimated depending on things like the person’s level of self-efficacy. Therefore, further studies are needed to study the association between subjective and objective eHealth literacy. Another limitation was the use of a nonvalidated question to assess general self-perceived health. However, it has been claimed that self-perceived health is one of the internationally leading health indicators reflecting a person’s subjective general perception of health [36]. It has also been argued that self-rated health is inclusive and dynamic in judging the trajectory of health and that it influences behaviors that subsequently affect health status and reflects resources that affect the ability to cope with health threats [37].

### Conclusion

This study confirmed that Sw-eHEALS is a reliable and valid tool for assessing the perceived comfort and skills of Swedish speakers in using information technology for health (ie, eHealth literacy). However, there are some linguistic issues relating to the concept of *health resources*. The adapted threshold levels for inadequate, problematic, and sufficient levels of eHealth literacy seem to be relevant and important when conducting further studies, especially intervention studies.

## Abbreviations

COSMIN: Consensus-Based Standards for the Selection of Health Measurement Instruments
eHealth: electronic health
eHEALS: eHealth Literacy Scale
HLS-EU-Q16: Health Literacy Survey European Questionnaire, 16-item
Sw-eHEALS: Swedish version of eHEALS
